# A new sequence related to the Euler–Mascheroni constant

**DOI:** 10.1186/s13660-018-1746-3

**Published:** 2018-06-28

**Authors:** Shanhe Wu, Gabriel Bercu

**Affiliations:** 1 Department of Mathematics, Longyan University, Longyan, P.R. China; 2 Department of Mathematics and Computer Sciences, “Dunărea de Jos” University of Galaţi, Galaţi, Romania

**Keywords:** 11Y60, 41A60, 41A25, Sequences, Euler–Mascheroni constant, Rate of convergence, Lower and upper bounds

## Abstract

In this paper, we provide a new quicker sequence convergent to the Euler–Mascheroni constant using an approximation of Padé type. Our sequence has a relatively simple form and higher speed of convergence. Moreover, we establish lower and upper bound estimates for the difference between the sequence and the Euler–Mascheroni constant.

## Introduction

The Euler–Mascheroni constant

$$ \gamma =0.5772156649015328\ldots $$

is one of the most famous constants in analysis and number theory. It is the limit of the sequence

1.1 $$ \gamma_{n}=1+\frac{1}{2}+\cdots +\frac{1}{n}-\log n. $$

There are many famous problems related to the properties of this constant; for example, it is not known yet whether the Euler–Mascheroni constant is a rational number. In recent years, many researchers made great efforts in the area of concerning the rate of convergence of the sequence $(\gamma_{n})_{n\geq 1}$ and establishing sequences converging faster to the Euler–Mascheroni constant *γ*.

We begin with a brief overview of the relevant research.

To reveal the speed of convergence of the sequence $(\gamma_{n})_{n \geq 1}$, Boas [5] and Mortici and Vernescu [20, 21] established the following double inequality for the difference between the sequence and the Euler–Mascheroni constant:

1.2 $$ \frac{1}{2n+1} < \gamma_{n}-\gamma < \frac{1}{2n}. $$

DeTemple [12] modified the logarithmic term of $\gamma_{n}$ and showed that the sequence

1.3 $$ R_{n}=1+\frac{1}{2}+\cdots +\frac{1}{n}-\log \biggl( n+ \frac{1}{2} \biggr) $$

converges to *γ* with rate of convergence $n^{-2}$, since

1.4 $$ \frac{1}{24(n+1)^{2}} < R_{n}-\gamma < \frac{1}{24n^{2}}. $$

Vernescu [28] provided the sequence

1.5 $$ V_{n}=1+\frac{1}{2}+\cdots +\frac{1}{n-1}+\frac{1}{2n}- \log n, $$

which also converges to *γ* with rate of convergence $n^{-2}$, since

1.6 $$ \frac{1}{12(n+1)^{2}}< \gamma -V_{n}< \frac{1}{12n^{2}}. $$

Cristea and Mortici [11] introduced the family of sequences

1.7 $$ v_{n}(a,b)=1+\frac{1}{2}+\cdots +\frac{1}{n-2}+ \frac{an+b}{n(n-1)}- \log n, $$

where *a*, *b* are real parameters. Furthermore, they proved that, among the sequences $(v_{n}(a,b))_{n\geq 1}$, the privileged one $( v _{n} ( \frac{3}{2},-\frac{5}{12} ) ) _{n\geq 1}$ offers the best approximation to *γ*, since it has the rate of convergence $n^{-3}$. More precisely, for

1.8 $$ v_{n} \biggl( \frac{3}{2},-\frac{5}{12} \biggr) =1+ \frac{1}{2}+\cdots +\frac{1}{n-2}+\frac{13}{12(n-1)}+ \frac{5}{12n}-\log n, $$

they obtained the bounds

1.9 $$ \frac{1}{12n^{3}}+\frac{11}{120n^{4}}< v_{n} \biggl( \frac{3}{2},- \frac{5}{12} \biggr) -\gamma < \frac{1}{12n^{3}}+ \frac{13}{120n^{4}} \quad (n\geq 9). $$

Lu [16] used continued fraction approximation to obtain the following faster sequence converging to the Euler–Mascheroni constant:

1.10 $$ r_{n}^{(2)}{ \biggl( \frac{1}{2},\frac{1}{6} \biggr) }=1+\frac{1}{2}+ \cdots +\frac{1}{n}-\frac{3}{6n+1}-\log n, $$

which satisfies

1.11 $$ \frac{1}{72(n+1)^{3}}< \gamma -r_{n}^{(2)}{ \biggl( \frac{1}{2}, \frac{1}{6} \biggr) }< \frac{1}{72n^{3}}. $$

Recently, Wu and Bercu [29] constructed the new sequence

1.12 $$ \omega_{n}=\sum_{k=1}^{n}{ \frac{1+(2b_{1}-1)(-1)^{k-1}}{k}}-\log \biggl[ n+{\frac{(-1)^{n-1}(2b_{1}-1)+1}{2}} \biggr] -(2b_{1}-1)\log 2, $$

which converges to *γ* with rate of convergence $n^{-2}$.

For more detail about the approximation of the Euler–Mascheroni constant with very high accuracy, we mention the works of Lu [16–18], Sweeney [27], Bailey [2], Crînganu [10], and Alzer and Koumandos [1]. We also mention the excellent survey by Lagarias [15]. Hu and Mortici [13, 14, 19] provided some similar methods to deal with approximation of the constant *e*.

In this paper, starting from the sequence $(\gamma_{n})_{n\geq 1}$, we use an approximation of Padé type and provide a new convergent sequence for Euler–Mascheroni constant.

The Padé approximant is the best approximation of a function by a rational function and often gives better approximation of the function than truncating its Taylor series. For these reasons, Padé approximants are also used in computer calculations (see [3, 30]).

Recall the Padé approximant of $P(n)$ of order $[1/2]$:

1.13 $$ P_{[1/2]}(n)=\frac{\alpha_{0}+\alpha_{1}n}{1+\beta_{1}n+\beta_{2}n ^{2}}=\frac{a_{1}}{n+b_{1}}+\frac{a_{2}}{n+b_{2}}. $$

We will use this Padé approximant $p_{[1/2]}(n)$ as an additional term to establish a new quicker sequence converging to the Euler–Mascheroni constant. More precisely, we consider the following sequence:

1.14 $$ \Gamma_{n}^{(2)}=1+\frac{1}{2}+\cdots + \frac{1}{n}-\log n-\frac{a _{1}}{n+b_{1}}-\frac{a_{2}}{n+b_{2}}. $$

Furthermore, we will provide lower and upper bound estimates for the difference between the sequence and the Euler–Mascheroni constant.

## Main results

Our main results are stated in the following theorem.

### Theorem 2.1

*Let*

$$ \Gamma_{n}^{(2)}=1+\frac{1}{2}+\cdots + \frac{1}{n}-\log n-\frac{a _{1}}{n+b_{1}}-\frac{a_{2}}{n+b_{2}}, $$

*and let*

$$\begin{aligned}& a_{1}=\frac{1}{24b_{1}(1-3b_{1})}, \\& a_{2}=-\frac{(6b_{1}-1)^{2}}{24b _{1}(1-3b_{1})}, \\& b_{2}=\frac{b_{1}}{6b_{1}-1},\quad b_{1}\in \biggl( \frac{1}{6},\frac{1}{3} \biggr)\cup \biggl(\frac{1}{3},+ \infty \biggr). \end{aligned}$$

*Then we have the asymptotic expansion*

2.1 $$ \Gamma_{n}^{(2)}=\gamma +\sum_{k=4}^{m} \biggl( -\frac{B_{k}}{k}+(-1)^{k} \bigl(a _{1}b_{1}^{k-1}+a_{2}b_{2}^{k-1} \bigr) \biggr) \frac{1}{n^{k}}+O \biggl( \frac{1}{n ^{m+1}} \biggr) $$

*as* $n\rightarrow \infty $, *where* $B_{k}$ *are Bernoulli numbers*. *More explicitly*, *we have*

2.2 $$\begin{aligned} \Gamma_{n}^{(2)}&=\gamma +\frac{1-10p}{120}\cdot \frac{1}{n^{4}}+\frac{p ^{2}}{2}\cdot \frac{1}{n^{5}}+ \biggl( \frac{p^{2}-36p^{3}}{12}- \frac{1}{252} \biggr) \frac{1}{n^{6}}+{p^{3}}(18p-1) \frac{1}{n^{7}} \\ &\quad {}+\cdots + \biggl( \frac{(3-\sqrt{9-p^{-1}})^{m-3}-(3+ \sqrt{9-p^{-1}})^{m-3}}{24(-1)^{m}p^{3-m}\sqrt{9-p^{-1}}}-\frac{B _{m}}{m} \biggr) \frac{1}{n^{m}}+O \biggl( \frac{1}{n^{m+1}} \biggr) \end{aligned}$$

*as* $n\rightarrow \infty $, *where* $p=b_{1}^{2}/(6b_{1}-1)$.

*Furthermore*, *we have the following double inequality*:

2.3 $$\begin{aligned} \frac{1-10p}{120}\cdot \frac{1}{n^{4}}< \Gamma_{n}^{(2)}- \gamma < \frac{1-10p}{120}\cdot \frac{1}{n^{4}}+\frac{p^{2}}{2}\cdot \frac{1}{n ^{5}}. \end{aligned}$$

### Proof

Using the representation of the harmonic sum in terms of digamma function (see [4])

2.4 $$ 1+\frac{1}{2}+\cdots +\frac{1}{n}=\gamma +\frac{1}{n}+\Psi (n) $$

and the asymptotic formula

2.5 $$\begin{aligned} \Psi (z)&=\log z-\frac{1}{2z}-\sum_{k=2}^{m} \frac{B_{2k-2}}{(2k-2)z ^{2k-2}}+O \biggl( \frac{1}{z^{2m}} \biggr) \\ &=\log z-\frac{1}{2z}-\frac{1}{12z^{2}}+\frac{1}{120z^{4}}- \frac{1}{252z ^{6}}+\cdots +\frac{-B_{2m-2}}{(2m-2)z^{2m-2}}+O \biggl( \frac{1}{z^{2m}} \biggr) , \end{aligned}$$

we obtain

$$\begin{aligned} &1+\frac{1}{2}+\cdots +\frac{1}{n}-\log n \\ &\quad =\gamma +\frac{1}{n}+ \Psi (n)- \log n \\ &\quad =\gamma +\frac{1}{2n}-\frac{1}{12n^{2}}+\frac{1}{120n^{4}}- \frac{1}{252n ^{6}}+\cdots +\frac{-B_{2m-2}}{(2m-2)n^{2m-2}}+O \biggl( \frac{1}{n^{2m}} \biggr) . \end{aligned}$$

Hence

$$\begin{aligned} \Gamma_{n}^{(2)} & =1+\frac{1}{2}+\cdots + \frac{1}{n}-\log n-\frac{a _{1}}{n+b_{1}}-\frac{a_{2}}{n+b_{2}} \\ & =\gamma -\frac{a_{1}}{n+b_{1}}-\frac{a_{2}}{n+b_{2}}+ \frac{1}{2n}- \frac{1}{12n^{2}}+\frac{1}{120n^{4}} \\ & \quad {}-\frac{1}{252n^{6}}+\cdots +\frac{-B_{2m-2}}{(2m-2)n^{2m-2}}+O \biggl( \frac{1}{n^{2m}} \biggr) . \end{aligned}$$

Using the power series expansion gives

$$\begin{aligned} \frac{a_{1}}{n+b_{1}} & =\frac{a_{1}}{n} \biggl( \frac{1}{1+\frac{b _{1}}{n}} \biggr) \\ &= \frac{a_{1}}{n} \biggl(1-\frac{b_{1}}{n}+\frac{b_{1}^{2}}{n ^{2}}- \frac{b_{1}^{3}}{n^{3}}+\cdots +(-1)^{m}\frac{b_{1}^{m}}{n^{m}} \biggr)+O \biggl( \frac{1}{n^{m+2}} \biggr) \\ & =\frac{a_{1}}{n}-\frac{a_{1}b_{1}}{n^{2}}+\frac{a_{1}b_{1}^{2}}{n ^{3}}-\frac{a_{1}b_{1}^{3}}{n^{4}}+ \cdots +(-1)^{m}\frac{a_{1}b_{1} ^{m}}{n^{m+1}}+O \biggl( \frac{1}{n^{m+2}} \biggr) \end{aligned}$$

and

$$ \frac{a_{2}}{n+b_{2}}=\frac{a_{2}}{n}-\frac{a_{2}b_{2}}{n^{2}}+\frac{a _{2}b_{2}^{2}}{n^{3}}- \frac{a_{2}b_{2}^{3}}{n^{4}}+\cdots +(-1)^{m}\frac{a _{2}b_{2}^{m}}{n^{m+1}}+O \biggl( \frac{1}{n^{m+2}} \biggr) $$

as $n\rightarrow \infty $. Thus we obtain

$$\begin{aligned} \Gamma_{n}^{(2)} & = \gamma + \biggl( \frac{1}{2}-a_{1}-a_{2} \biggr) \frac{1}{n}+ \biggl( - \frac{1}{12}+a_{1}b_{1}+a_{2}b_{2} \biggr) \frac{1}{n^{2}}- \bigl( a _{1}b_{1}^{2}+a_{2}b_{2}^{2} \bigr) \frac{1}{n^{3}} \\ & \quad {}+ \biggl( \frac{1}{120}+a_{1}b_{1}^{3}+a_{2}b_{2}^{3} \biggr) \frac{1}{n ^{4}}- \bigl( a_{1}b_{1}^{4}+a_{2}b_{2}^{4} \bigr) \frac{1}{n^{5}}+ \biggl( -\frac{1}{252}+a_{1}b_{1}^{5}+a_{2}b_{2}^{5} \biggr) \frac{1}{n ^{6}} \\ & \quad {} +\cdots + \biggl( \frac{-B_{2m-2}}{2m-2}+a_{1}b_{1}^{2m-3}+a_{2}b _{2}^{2m-3} \biggr) \frac{1}{n^{2m-2}} \\ & \quad {} + \bigl( -a_{1}b_{1}^{2m-2}-a_{2}b_{2}^{2m-2} \bigr) \frac{1}{n^{2m-1}}+O \biggl( \frac{1}{n^{2m}} \biggr) . \end{aligned}$$

From the assumption conditions

$$\begin{aligned}& a_{1}=\frac{1}{24b_{1}(1-3b_{1})}, \\& a_{2}=-\frac{(6b_{1}-1)^{2}}{24b _{1}(1-3b_{1})}, \\& b_{2}=\frac{b_{1}}{6b_{1}-1},\quad b_{1}\in \biggl( \frac{1}{6},\frac{1}{3} \biggr)\cup \biggl(\frac{1}{3},+ \infty \biggr), \end{aligned}$$

we have

$$ \frac{1}{2}-a_{1}-a_{2}=0,\qquad -\frac{1}{12}+a_{1}b_{1}+a_{2}b_{2}=0,\qquad a_{1}b_{1}^{2}+a_{2}b_{2}^{2}=0. $$

Therefore

$$\begin{aligned} \Gamma_{n}^{(2)}&=\gamma+ \biggl( \frac{1}{120}+a_{1}b_{1}^{3}+a_{2}b_{2}^{3} \biggr) \frac{1}{n ^{4}}- \bigl( a_{1}b_{1}^{4}+a_{2}b_{2}^{4} \bigr) \frac{1}{n^{5}}+ \biggl( -\frac{1}{252}+a_{1}b_{1}^{5}+a_{2}b_{2}^{5} \biggr) \frac{1}{n ^{6}} \\ & \quad {}+\cdots + \biggl( \frac{-B_{2m-2}}{2m-2}+a_{1}b_{1}^{2m-3}+a_{2}b _{2}^{2m-3} \biggr) \frac{1}{n^{2m-2}} \\ & \quad {}+ \bigl(-a_{1}b_{1}^{2m-2}-a_{2}b_{2}^{2m-2} \bigr)\frac{1}{n^{2m-1}}+O \biggl( \frac{1}{n ^{2m}} \biggr) . \end{aligned}$$

Note that, for all odd Bernoulli numbers $B_{2m-1}=0$ $(m\geq 2)$, the last expression can be rewritten as

$$\begin{aligned} \Gamma_{n}^{(2)}&=\gamma+ \biggl( \frac{1}{120}+a_{1}b_{1}^{3}+a_{2}b_{2}^{3} \biggr) \frac{1}{n ^{4}}+\cdots + \biggl( \frac{-B_{2m-2}}{2m-2}+a_{1}b_{1}^{2m-3}+a_{2}b _{2}^{2m-3} \biggr) \frac{1}{n^{2m-2}} \\ & \quad {} + \biggl( \frac{-B_{2m-1}}{2m-1}-a_{1}b_{1}^{2m-2}-a_{2}b_{2}^{2m-2} \biggr) \frac{1}{n ^{2m-1}}+O \biggl( \frac{1}{n^{2m}} \biggr) \end{aligned}$$

and

$$\begin{aligned} \Gamma_{n}^{(2)} &=\gamma + \biggl(\frac{1}{120}+a_{1}b_{1}^{3}+a_{2}b _{2}^{3} \biggr)\frac{1}{n^{4}}+\cdots + \biggl( \frac{-B_{2m-3}}{2m-3}+a _{1}b_{1}^{2m-4}+a_{2}b_{2}^{2m-4} \biggr)\frac{1}{n^{2m-3}} \\ & \quad {}+ \biggl(\frac{-B_{2m-2}}{2m-2}+a_{1}b_{1}^{2m-3}+a_{2}b_{2}^{2m-3} \biggr)\frac{1}{n^{2m-2}}+O \biggl(\frac{1}{n^{2m-1}} \biggr), \end{aligned}$$

that is,

2.6 $$ \Gamma_{n}^{(2)}=\gamma +\sum_{k=4}^{m} \biggl( -\frac{B_{k}}{k}+(-1)^{k} \bigl(a _{1}b_{1}^{k-1}+a_{2}b_{2}^{k-1} \bigr) \biggr) \frac{1}{n^{k}}+O \biggl( \frac{1}{n ^{m+1}} \biggr) , $$

which is the desired Eq. (2.1) in Theorem 2.1.

On the other hand, from

$$ p=\frac{b_{1}^{2}}{6b_{1}-1},\qquad b_{2}=\frac{b_{1}}{6b_{1}-1},\quad b_{1}\in \biggl(\frac{1}{6},\frac{1}{3} \biggr)\cup \biggl(\frac{1}{3},+ \infty \biggr), $$

we have $b_{1}b_{2}=p$, $b_{1}+b_{2}=6p$ ($p>\frac{1}{9}$), which implies that $b_{1}$ and $b_{2}$ are the roots of the equation $x^{2}-6px+p=0$. Therefore,

$$ b_{1,2}=3p\pm \sqrt{9p^{2}-p}. $$

It is easy to observe that

$$\begin{aligned} a_{1} & =\frac{1}{24b_{1}(1-3b_{1})}=\frac{b_{2}}{12b_{1}(b_{2}-b _{1})}, \\ a_{2} & =-\frac{(6b_{1}-1)^{2}}{24b_{1}(1-3b_{1})}=\frac{-b_{1}}{12b _{2}(b_{2}-b_{1})}, \end{aligned}$$

and thus

$$\begin{aligned} a_{1}b_{1}^{k-1}+a_{2}b_{2}^{k-1} & =\frac{b_{2}b_{1}^{k-1}}{12b_{1}(b _{2}-b_{1})}-\frac{b_{1}b_{2}^{k-1}}{12b_{2}(b_{2}-b_{1})} \\ & =-\frac{b_{1}b_{2}(b_{1}^{k-3}-b_{2}^{k-3})}{12(b_{1}-b_{2})} \\ & =\frac{(3-\sqrt{9-p^{-1}})^{k-3}-(3+\sqrt{9-p^{-1}})^{k-3}}{24p ^{3-k}\sqrt{9-p^{-1}}}. \end{aligned}$$

It follows from (2.6) that

$$\begin{aligned} \Gamma_{n}^{(2)}& =\gamma +\sum_{k=4}^{m} \biggl( -\frac{B_{k}}{k}+(-1)^{k} \bigl(a _{1}b_{1}^{k-1}+a_{2}b_{2}^{k-1} \bigr) \biggr) \frac{1}{n^{k}}+O \biggl( \frac{1}{n ^{m+1}} \biggr) \\ & =\gamma +\sum_{k=4}^{m} \biggl( - \frac{B_{k}}{k}+\frac{(3-\sqrt{9-p ^{-1}})^{k-3}-(3+\sqrt{9-p^{-1}})^{k-3}}{24(-1)^{k}p^{3-k}\sqrt{9-p ^{-1}}} \biggr) \frac{1}{n^{k}}+O \biggl( \frac{1}{n^{m+1}} \biggr) , \end{aligned}$$

which implies the desired Eq. (2.2) in Theorem 2.1.

Next, we will show the double inequality (2.3). We define the sequences $(z_{n})_{n\geq 1}$ and $(u_{n})_{n\geq 1}$ by

$$ z_{n}=\Gamma_{n}^{(2)}-\gamma -\frac{1-10p}{120} \cdot \frac{1}{n^{4}} $$

and

$$ u_{n}=\Gamma_{n}^{(2)}-\gamma -\frac{1-10p}{120} \cdot \frac{1}{n ^{4}} -\frac{p^{2}}{2}\cdot \frac{1}{n^{5}}. $$

It follows from (2.2) that

$$\begin{aligned}& \Gamma_{n}^{(2)}-\gamma -\frac{1-10p}{120}\cdot \frac{1}{n^{4}}=O \biggl( \frac{1}{n^{5}} \biggr) , \\& \Gamma_{n}^{(2)}-\gamma -\frac{1-10p}{120}\cdot \frac{1}{n^{4}}-\frac{p ^{2}}{2}\cdot \frac{1}{n^{5}}=O \biggl( \frac{1}{n^{6}} \biggr) , \end{aligned}$$

and thus we have

$$ \lim_{n\rightarrow \infty }z_{n}=0\quad \text{and }\quad \lim _{n\rightarrow \infty }u_{n}=0. $$

To prove that $z_{n}>0$ and $u_{n}<0$ for $n\geq 1$, it suffices to show that $(z_{n})_{n\geq 1}$ is decreasing and $(u_{n})_{n\geq 1}$ is increasing.

Let

$$\begin{aligned} z_{n+1}-z_{n} & =f(n), \\ u_{n+1}-u_{n} & =g(n), \end{aligned}$$

where

$$\begin{aligned}& f(x) =\frac{1}{x+1}+\log x-\log (x+1)+a_{1} \biggl( \frac{1}{x+b _{1}}-\frac{1}{x+b_{1}+1} \biggr) \\& \hphantom{f(x) =}{}+a_{2} \biggl( \frac{1}{x+b_{2}}-\frac{1}{x+b_{2}+1} \biggr) + \frac{10p-1}{120} \biggl( \frac{1}{(x+1)^{4}}-\frac{1}{x^{4}} \biggr) , \\& g(x)=f(x)-\frac{p^{2}}{2} \biggl( \frac{1}{(x+1)^{5}}- \frac{1}{x^{5}} \biggr) ,\quad x\in {}[ 1,+\infty ). \end{aligned}$$

It is easy to verify that

$$ \frac{a_{1}}{x+b_{1}}+\frac{a_{2}}{x+b_{2}}=\frac{6x+36p-1}{12(x ^{2}+6px+p)} $$

and

$$ \frac{a_{1}}{x+b_{1}+1}+\frac{a_{2}}{x+b_{2}+1}=\frac{6x+36p+5}{12(x ^{2}+2x++6px+1+7p)}. $$

Hence

$$\begin{aligned} f(x) & =\frac{1}{x+1}+\log x-\log (x+1)+ \frac{6x+36p-1}{12(x^{2}+6px+p)} \\ & \quad {}-\frac{6x+36p+5}{12(x^{2}+ 2x++6px+1+7p)}+\frac{10p-1}{120} \biggl( \frac{1}{(x+1)^{4}}- \frac{1}{x ^{4}} \biggr) . \end{aligned}$$

Differentiating $f(x)$ with respect to *x* gives

$$ f^{\prime }(x)=\frac{P(x)}{30x^{5} ( x+1 ) ^{5} ( x^{2}+6px+p ) ^{2} ( x ^{2}+ 2x+6px+1+7p ) ^{2}}, $$

where

$$\begin{aligned} P(x)& =450p^{2}x^{11}+ \biggl( 7020p^{2} \biggl( p-\frac{1}{9} \biggr)+3360p \biggl( p-\frac{1}{9} \biggr) + \frac{3360}{9} \biggl( p-\frac{1}{9} \biggr) +\frac{2955}{81} \biggr) x^{10} \\ & \quad {}+ \biggl( 36\text{,}720p^{3} \biggl( p-\frac{1}{9} \biggr) +41\text{,}820p^{2} \biggl( p- \frac{1}{9} \biggr) +\frac{102\text{,}570}{9}p \biggl( p-\frac{1}{9} \biggr) \\ & \quad {} +\frac{92\text{,}850}{81} \biggl( p-\frac{1}{9} \biggr) + \frac{74\text{,}625}{729} \biggr) x^{9}+ \biggl( 64\text{,}800p^{4} \biggl( p- \frac{1}{9} \biggr) +191\text{,}520p^{3} \biggl( p-\frac{1}{9} \biggr) \\ & \quad {} +\frac{1\text{,}018\text{,}260}{9}p^{2} \biggl( p- \frac{1}{9} \biggr) + \frac{1\text{,}798\text{,}290}{81}p \biggl( p-\frac{1}{9} \biggr) + \frac{1\text{,}390\text{,}050}{729} \biggl( p-\frac{1}{9} \biggr) \\ &\quad {}+ \frac{1\text{,}055\text{,}439}{6561} \biggr) x^{8} \\ & \quad {} + \biggl( 302\text{,}400p^{4} \biggl( p-\frac{1}{9} \biggr) +446\text{,}910p^{3} \biggl( p-\frac{1}{9} \biggr) + \frac{1\text{,}619\text{,}250}{9}p^{2} \biggl( p-\frac{1}{9} \biggr) \\ & \quad {} +\frac{2\text{,}158\text{,}710}{81}p \biggl( p-\frac{1}{9} \biggr) + \frac{1\text{,}383\text{,}054}{729} \biggl( p-\frac{1}{9} \biggr) + \frac{1\text{,}028\text{,}760}{6561} \biggr) x^{7} \\ & \quad {} + \biggl( 615\text{,}600p^{4} \biggl( p-\frac{1}{9} \biggr) +612\text{,}290p^{3} \biggl( p-\frac{1}{9} \biggr) + \frac{1\text{,}632\text{,}350}{9}p^{2} \biggl( p-\frac{1}{9} \biggr) \\ & \quad {} +\frac{1\text{,}634\text{,}294}{81}p \biggl( p-\frac{1}{9} \biggr) + \frac{863\text{,}012}{729} \biggl( p-\frac{1}{9} \biggr) + \frac{659\text{,}621}{6561} \biggr) x^{6} \\ & \quad {} + \biggl( 724\text{,}800p^{4} \biggl( p-\frac{1}{9} \biggr)+ \frac{4\text{,}803\text{,}690}{9}p^{3} \biggl( p-\frac{1}{9} \biggr) + \frac{9\text{,}579\text{,}126}{81}p^{2} \biggl( p-\frac{1}{9} \biggr) \\ & \quad {} +\frac{7\text{,}355\text{,}676}{729}p \biggl( p-\frac{1}{9} \biggr) + \frac{3\text{,}484\text{,}686}{6561} \biggl( p-\frac{1}{9} \biggr) + \frac{2\text{,}953\text{,}245}{59\text{,}049} \biggr) x^{5} \\ & \quad {} + \biggl( 536\text{,}210p^{4} \biggl( p-\frac{1}{9} \biggr) + \frac{2\text{,}700\text{,}431}{9}p^{3} \biggl( p-\frac{1}{9} \biggr) + \frac{4\text{,}053\text{,}779}{81}p^{2} \biggl( p-\frac{1}{9} \biggr) \\ & \quad {}+\frac{2\text{,}169\text{,}314}{729}p \biggl( p-\frac{1}{9} \biggr) + \frac{962\text{,}090}{6561} \biggl( p-\frac{1}{9} \biggr) + \frac{903\text{,}041}{59\text{,}049} \biggr) x^{4} \\ & \quad {} + \biggl( 247\text{,}060p^{4} \biggl( p-\frac{1}{9} \biggr) +\frac{968\text{,}266}{9}p ^{3} \biggl( p-\frac{1}{9} \biggr) + \frac{1\text{,}113\text{,}742}{81}p^{2} \biggl( p- \frac{1}{9} \biggr) \\ & \quad {} +\frac{437\text{,}230}{729}p \biggl( p-\frac{1}{9} \biggr) +\frac{240\text{,}400}{6561} \biggl( p-\frac{1}{9} \biggr) +\frac{240\text{,}400}{59\text{,}049} \biggr) x^{3}+ \biggl( 66\text{,}580p^{4} \biggl( p-\frac{1}{9}\biggr) \\ & \quad {} +\frac{213\text{,}658}{9}p^{3} \biggl( p- \frac{1}{9} \biggr) + \frac{195\text{,}838}{81}p^{2} \biggl( p- \frac{1}{9} \biggr) + \frac{58\text{,}786}{729}p \biggl( p-\frac{1}{9} \biggr) \\ &\quad {}+\frac{45\text{,}664}{6561} \biggl( p-\frac{1}{9} \biggr)+ \frac{45\text{,}664}{59\text{,}049} \biggr) x^{2} \\ & \quad {} + \biggl(9170p^{4} \biggl( p- \frac{1}{9} \biggr) +\frac{25\text{,}937}{9}p^{3} \biggl( p-\frac{1}{9} \biggr) +\frac{20\text{,}429}{81}p^{2} \biggl( p-\frac{1}{9} \biggr) + \frac{5120}{729}p^{2} \biggr) x \\ & \quad {} + \biggl( 490p^{4} \biggl( p-\frac{1}{9} \biggr) +\frac{1309}{9}p^{3} \biggl( p-\frac{1}{9} \biggr) +\frac{985}{81}p^{2} \biggl( p- \frac{1}{9} \biggr) +\frac{256}{729}p^{2} \biggr) . \end{aligned}$$

Since $p>\frac{1}{9}$, we have $P(x)>0$, which implies that $f^{\prime }(x)>0$ for ${{x\in {}[ 1,+\infty ).}}$ Hence $f(x)$ is strictly increasing on $[1,+\infty )$. It follows from $\lim_{x\rightarrow +\infty }f(x)=0$ that $f(x)<0$ for ${{x\in {}[ 1,+\infty ).}}$ This yields $z_{n+1}-z_{n}=f(n)<0$, so that $(z_{n})_{n\geq 1}$ is strictly decreasing, which, along with $\lim_{n\rightarrow \infty }z_{n}=0$, leads us to $z_{n}>0$. The left-hand inequality of (2.3) is proved.

Similarly, differentiating $g(x)$ with respect to *x*, we obtain

$$ g^{\prime }(x)=\frac{-Q(x)}{ 30x^{6} ( x+1 ) ^{6} ( x^{2}+6px+p ) ^{2} ( x ^{2}+ 2x+6px+1+7p ) ^{2} }, $$

where

$$\begin{aligned} Q(x) & = \biggl( 3780p^{2} \biggl( p-\frac{1}{9} \biggr) + \frac{2835}{9}p ^{2}+5 \biggr) x^{12}+60\text{,}480p^{3} \biggl( p-\frac{1}{9} \biggr) \\ & \quad {} +28\text{,}560p^{2} \biggl( p-\frac{1}{9} \biggr) + \frac{22\text{,}890}{9}p ^{2}+120p+30 ) x^{11}+ \biggl( 324\text{,}000p^{4} \biggl( p-\frac{1}{9} \biggr) \\ & \quad {} +381\text{,}960p^{3} \biggl( p-\frac{1}{9} \biggr) + \frac{916\text{,}560}{9}p ^{2} \biggl( p-\frac{1}{9} \biggr) + \frac{859\text{,}455}{81}p^{2}+680p \\ & \quad {} +76 \biggr) x^{10}+ \bigl( 583\text{,}200p^{6}+1\text{,}771\text{,}200p^{5}+927\text{,}870p ^{4}+105\text{,}180p^{3} \\ & \quad {} +2610p^{2}+1624p+105 \bigr) x^{9}+ \bigl( 3\text{,}013\text{,}200p^{6}+4\text{,}492\text{,}800p ^{5} \\ & \quad {} +1\text{,}555\text{,}150p^{4}+138\text{,}450p^{3}+9066p^{2}+2122p+85 \bigr) x^{8}+ \bigl( 7\text{,}095\text{,}600p^{6} \\ & \quad {} +7\text{,}020\text{,}600p^{5}+1\text{,}794\text{,}700p^{4}+138\text{,}504p^{3}+12\text{,}426p^{2}+1648p \\ & \quad {} +40 \bigr) x^{7}+ \bigl( 10\text{,}103\text{,}400p^{6}+7\text{,}393\text{,}390p^{5}+1\text{,}467\text{,}221p ^{4}+100\text{,}436p^{3} \\ & \quad {} +9010p^{2}+774p+10 \bigr) x^{6}+ \bigl( 9\text{,}461\text{,}250p^{6}+5\text{,}365\text{,}230p ^{5}+846\text{,}847p^{4} \\ & \quad {} +49\text{,}296p^{3}+4263p^{2}+214p+1 \bigr) x^{5}+ \bigl( 5\text{,}874\text{,}525p ^{6}+2\text{,}667\text{,}310p^{5} \\ & \quad {} +337\text{,}249p^{4}+15\text{,}224p^{3}+1191p^{2}+32p \bigr) x^{4}+ \bigl( 2\text{,}352\text{,}300p ^{6}+883\text{,}050p^{5} \\ & \quad {} +89\text{,}495p^{4}+2688p^{3}+209p^{2}+2p \bigr) x^{3}+ \bigl( 568\text{,}125p ^{6}+183\text{,}690p^{5} \\ & \quad {} +15\text{,}221p^{4}+222p^{3}+22p^{2} \bigr) x^{2}+ \bigl( 72\text{,}450p^{6}+21\text{,}410p ^{5}+1559p^{4} \\ & \quad {} +4p^{3}+p^{2} \bigr)x+ \bigl(3675p^{6}+1050p^{5}+75p^{4} \bigr) . \end{aligned}$$

Since $p>\frac{1}{9}$, we conclude that ${{Q}}(x)<0$. Thus we have $g{{^{\prime }}}$$(x)<0$ for ${{x\in {}[ 1,+\infty ).}}$ It follows that *g*$(x)$ is strictly decreasing on $[1,+\infty )$. Since $\lim_{x\rightarrow +\infty }g(x)=0$, we have $g(x)>0$ for ${{x\in {}[ 1,+\infty ).}}$ This yields $u_{n+1}-u_{n}=g(n)>0$, which implies that $(u_{n})_{n\geq 1}$ is strictly increasing. We obtain $u_{n}<0$ since $\lim_{n\rightarrow \infty }u_{n}=0$. The right-hand inequality of (2.3) is proved.

This completes the proof of Theorem 2.1. □

## Some remarks on Theorem 2.1

### Remark 3.1

Lu [16] constructed the sequence

3.1 $$ r_{n}^{(3)}=1+\frac{1}{2}+\cdots +\frac{1}{n}- \log n-\frac{a_{1}}{n+\frac{a _{2}\cdot n}{n+a_{3}}}, $$

where $a_{1}=\frac{1}{2}$, $a_{2}=\frac{1}{6}$, and $a_{3}=- \frac{1}{6}$ and proved the inequality

3.2 $$ \frac{1}{120(n+1)^{4}}< r_{n}^{(3)}-\gamma < \frac{1}{120(n-1)^{4}}. $$

In Theorem 2.1, if we take $p=\frac{1}{5}$ in inequality (2.3), then we get

3.3 $$ \frac{1}{120n^{4}}-\frac{1}{50n^{5}}< \gamma -\Gamma_{n}^{(2)}< \frac{1}{120n ^{4}}. $$

Since

$$ \frac{1}{120(n+1)^{4}}< \frac{1}{120n^{4}}-\frac{1}{50n^{5}} $$

and

$$ \frac{1}{120n^{4}}< \frac{1}{120(n-1)^{4}} $$

for all natural numbers $n\geq 5$, the sequence $( \Gamma_{n} ^{(2)} ) _{n\geq 1}$ provides a more accurate double inequality for the difference between the sequence and the Euler–Mascheroni constant than the sequence $( r_{n}^{(3)} ) _{n\geq 1}$ from [16].

### Remark 3.2

Lu et al. [18] considered the following sequence converging to the Euler–Mascheroni constant:

3.4 $$ r_{n,2}^{(3)}=1+\frac{1}{2}+\cdots +\frac{1}{n}- \log n-\frac{1}{2} \log \biggl( 1+\frac{a_{1}}{n+\frac{a_{2}\cdot n}{n+a_{3}}} \biggr) , $$

where $a_{1}=1$, $a_{2}=-\frac{1}{3}$, and $a_{3}=\frac{1}{3}$, and they proved that

3.5 $$ \frac{1}{180(n+1)^{4}}< \gamma -r_{n,2}^{(3)}< \frac{1}{180n^{4}}. $$

In Theorem 2.1, if we choose $p=\frac{1}{6}$ in inequality (2.3), then we obtain

3.6 $$ \frac{1}{180n^{4}}-\frac{1}{72n^{5}}< \gamma -\Gamma_{n}^{(2)}< \frac{1}{180n ^{4}}. $$

It is easy to find that

$$ \frac{1}{180(n+1)^{4}}< \frac{1}{180n^{4}}-\frac{1}{72n^{5}} $$

for all natural numbers $n\geq 5$, so the sequence $( \Gamma_{n} ^{(2)} ) _{n\geq 1}$ improves inequality (3.5) from [18].

### Remark 3.3

For more results relating to the Euler constant, sequences, and some estimates, we refer the interested reader to Sîntǎmǎrian [23–26] and the references therein.

## Conclusion

To provide a sequence converging faster to the Euler–Mascheroni constant, we construct a sequence $\Gamma_{n}^{(2)}$ by reference to the Padé approximant method, which improves the rate of convergence of the sequences introduced by Lu [16, 18]. Our sequence depends on a real parameter and has a relatively simple form. It is worth noting that the method mentioned is also applicable to establishing estimates of bounds for some special means. For example, the method can be used for further study on the results obtained previously by Chu et al. [6–9], Qian and Chu [22], Yang et al. [31–34], and Zhao et al. [35].
